# Lung Metastasis from Perineal Leiomyosarcoma: A Case Report and a Review of the Japanese Literature

**DOI:** 10.1155/2013/496304

**Published:** 2013-03-05

**Authors:** Masamichi Itoga, Wataru Ito, Shigeharu Ueki, Masahide Takeda, Yuki Moritoki, Yoshiki Kobayashi, Mami Chihara, Naoshi Suzuki, Hideto Sasaki, Junichi Chihara

**Affiliations:** ^1^Department of Infection, Allergy, Clinical Immunology and Laboratory Medicine, Akita University Graduate School of Medicine, 1-1-1, Hondo, Akita 010-8543, Japan; ^2^Senboku-Tazawa Municipal Hospital, Akita, Japan

## Abstract

Pulmonary metastasis from leiomyosarcoma is rare and its clinical management is challenging. A single lung metastasis from a perineal leiomyosarcoma occurred in a 79-year-old woman. Five months after resection of the lung metastasis, a new metastatic tumor developed in the contralateral lung. Since the patient did not desire to receive hospitalized treatment, TS-1 (an oral agent consisting of a combination of tegafur, gimeracil, and oteracil potassium) therapy was started on an outpatient basis. The lung metastasis has been successfully controlled for at least 17 months with excellent tolerability. The clinical features and the treatment of this case are discussed.

## 1. Introduction

Leiomyosarcoma, a relatively rare malignant tumor of smooth muscle origin, can arise from the wall of the gastrointestinal tract, wall of the uterus, soft tissues (e.g., subcutaneous tissue, deeper tissues, mesentery, and retroperitoneum) that are rich in a smooth muscle, or wall of the great vessels [1]. In general, middle-aged and older people are predominantly affected. While these tumors from the retroperitoneum and inferior vena cava have been reported to be more common in women, the male-female ratio has not been reported for tumors arising from other locations [2]. The tumors arise most frequently from the uterus, gastrointestinal tract, and retroperitoneal tissue including the pelvic cavity. Primary leiomyosarcomas of the rectum, external genitals, vagina, and perineal skin tissue are rarely reported [3].

Leiomyosarcomas are considered to metastasize more commonly via the hematogenous route [4]. The time interval to detection of metastasis is typically short, although some reports indicate diversity. At present, there is no standard chemotherapy for recurrent leiomyosarcoma. Nonaggressive chemotherapy might be useful for cases with intractable disease and/or of advanced age in terms of maintaining the patient's quality of life (QOL). We report a case with a lung metastasis from a perineal leiomyosarcoma that has been maintained for a long period of tumor dormancy with TS-1 (an oral fluoropyrimidine derivative consisting of tegafur, gimeracil, and oteracil) therapy.

## 2. Case Report

A 79-year-old woman visited our hospital because of an abnormal shadow on chest X-ray performed during a routine medical checkup in October 2009. She had no particular symptom. She had a medical history of uterine myoma in 1975, which was surgically removed by hysterectomy. She also had a medical history of perineal leiomyosarcoma in 2001. The tumor size was 3 × 2 cm and there were no lymph node metastases or remote metastasis. The disease stage was stage I according to Unio Internationalis Contra Cancrum (UICC). The tumor was totally removed and she had no chemotherapy. She had no family history of significant diseases.

**Figure 1:**
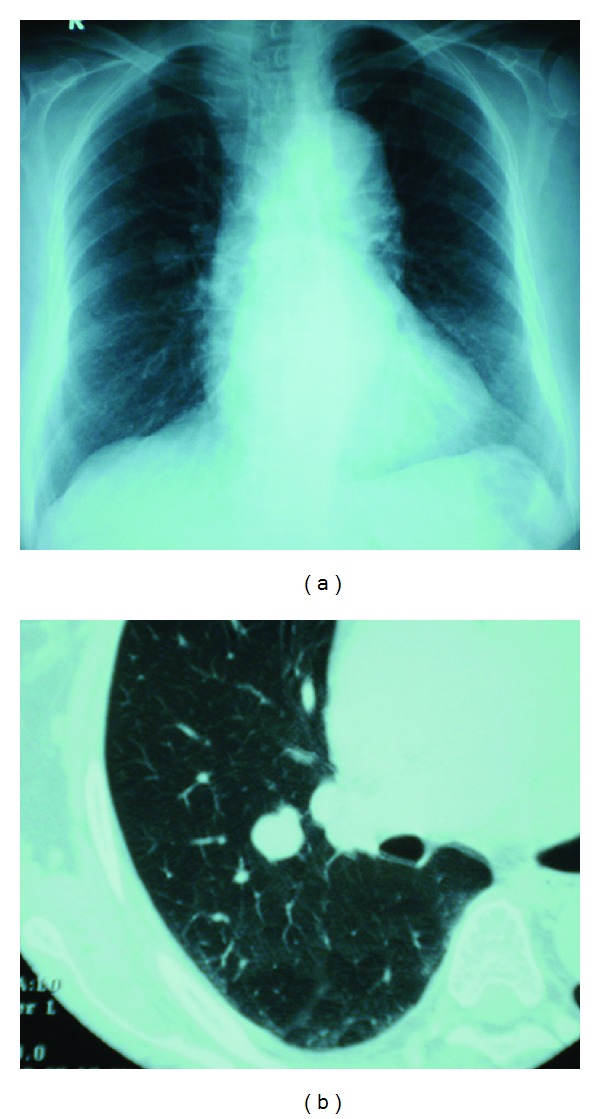
(a) Simple chest X-ray indicated a coin lesion on the middle field of the right lung. (b) Simple chest computed tomography revealed a nodule measuring 20 mm in diameter and having a well-defined border in S2 of the right lung.

On physical examination, her temperature was 35.8°C, blood pressure 119/78 mmHg, and pulse 68 beats per minute. The oxygen saturation was 97% with ambient air. Physical examination and laboratory tests (Table 1) showed no abnormalities. All tumor markers including squamous cell carcinoma antigen (SCC) and Sialyl Lewis X-i (SLX) showed no abnormalities. Simple chest radiography indicated a coin lesion on the middle field of the right lung (Figure 1(a)). Simple computed tomography (CT) of the chest revealed a nodule measuring 20 mm in diameter and having a well-defined border in area S2 of the right lung (Figure 1(b)). Thoracoscopic resection of the right upper lobe was performed in February 2010. Histopathological examination of the resected lesion revealed features consistent with leiomyosarcoma; therefore, based on the past medical history and histological similarity, the patient was diagnosed as having a lung metastasis from the peripapillary leiomyosarcoma.

**Figure 2:**
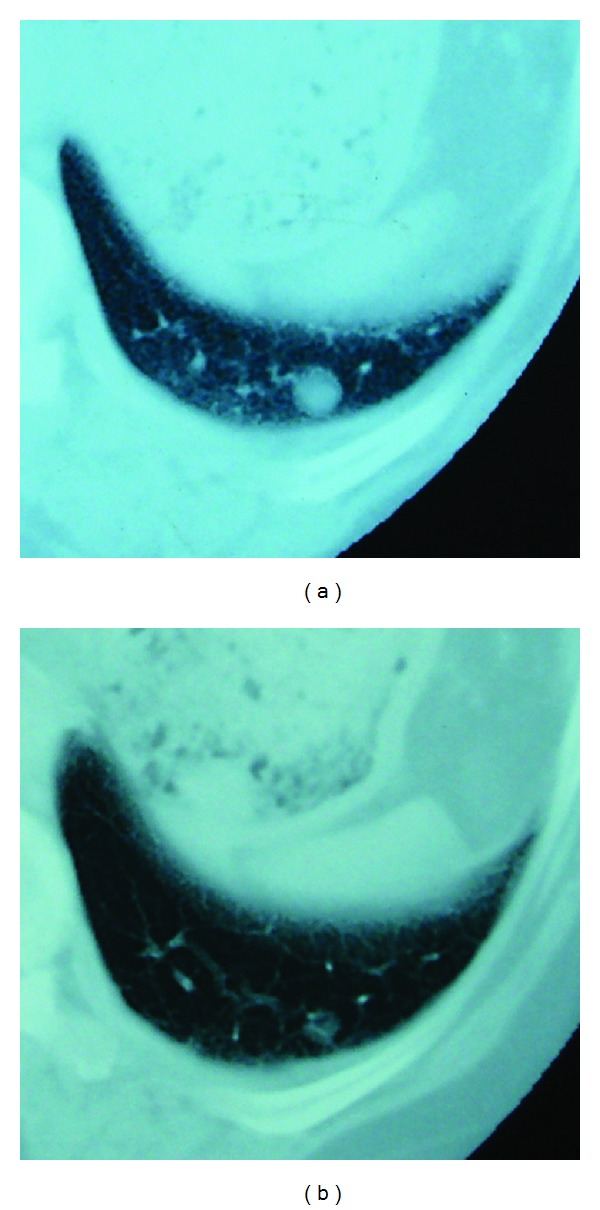
(a) In July 2010, a simple CT scan of the chest revealed a nodule with an irregular but well-defined margin in S10 of the left lung. (b) A chest CT scan obtained at the end of the second cycle of treatment revealed a reduction in tumor size.

Although she had been in her normal state of health after surgery, another tumor was detected in the opposite lung in July 2010. Simple CT of the chest revealed a nodule with an irregular but well-defined margin in S10 of the left lung (Figure 2(a)), and the clinical diagnosis of metastatic leiomyosarcoma was made. We suggested that she should be admitted to the hospital to receive combination chemotherapy, but she wanted to receive alternative therapy without hospitalization. Informed consent was obtained, and treatment with TS-1 on an outpatient basis was started in September 2010. The first cycle of treatment consisted of TS-1 100 mg/day administered in two divided doses for 28 days, followed by a treatment-free interval for 14 days. Since the patient reported loss of appetite after the end of the treatment, the TS-1 dose was reduced to 80 mg/day for the second cycle. A chest CT scan obtained at the end of the second cycle of treatment revealed a reduction in tumor size (Figure 2(b)). Subsequently, from the fourth cycle of treatment, TS-1 was administered at 80 mg/day for 14 days, followed by a drug-free period of 14 days. A total of 16 cycles of treatment were completed in March 2012. The patient remains free of side effects and shows no enlargement of the tumor. Her performance status was maintained.

## 3. Discussion

According to our literature search of the past decade in Japan, the lung was the first metastasis site in 53 cases of leiomyosarcoma. The ratio of females to males was approximately 3 to 1. The median age in the 53 cases was 58 years old (range, 34–89 years). The primary lesion was in the uterus in 31 cases, in the urinary bladder in three cases, in the thigh in two cases, and in the inferior vena cava in two cases. Perineal leiomyosarcoma has not been reported as the primary lesion. The mean interval from diagnosis of the primary tumor to detection of the pulmonary metastasis was 31 months (0 day to 12 years); in 11 cases, the metastasis was detected over 5 years after diagnosis of the primary tumor. Some cases were diagnosed as leiomyosarcoma with metastatic lesions due to the rapidly developing metastasis. The mean survival after diagnosis of the lung metastasis was 18 months (range, 26 days to 10 years). The present case's interval to the diagnosis of lung metastasis was relatively long, that is, 8 years from resection of the primary tumor. Because of the varied prognosis of leiomyosarcoma, patients should be closely monitored for a long period even if the primary lesion was surgically removed.

According to a previous report on the growth rate of metastasis from leiomyosarcomas of the stomach, the doubling time was 20 months or less [5]. Uterine leiomyosarcomas often follow a rapid clinical course and account for only 1% or less of uterine malignancies. Metastatic leiomyosarcomas have been treated with surgery and/or adjuvant chemotherapy, but no standard treatment has been established due to the small number of patients. Various combinations of antineoplastic drugs have been used, and a number of trials have been performed to establish a standard chemotherapy regimen for recurrent leiomyosarcomas. A recent phase II study of docetaxel + gemcitabine (DG treatment) [6] and second-line treatment with DG [7] were found to yield no significant survival benefit for leiomyosarcomas. We conducted a search of the literature for reports of cases treated with chemotherapy for lung metastases from leiomyosarcoma in the past decade in Japan (Table 2).

TS-1 is a fluoropyrimidine derivative in which tegafur is combined with two 5-FU-modulating substances, 5-chloro-2,4-dihydroxypyridine (gimeracil), and oteracil potassium, at a molecular ratio of 1 : 0.4 : 1. TS-1 was invented by Shirasaka et al. in 1991, and is the first self-rescuing concept-based anticancer agent [20]. Although myelosuppression is the main toxic effect induced by TS-1, administration of TS-1 causes a low incidence of blood toxicity. Further, TS-1 can be administered orally, which permits treatment on an outpatient basis, improving a patient's QOL [21]. TS-1 has been mainly used for treatment of stomach cancer, colon and rectal cancer, head and neck cancer, non-small-cell lung cancer, inoperability or recurrence of breast cancer, pancreatic cancer, and biliary cancer, and also for leiomyosarcoma in some cases [22, 23]. Chuganji et al. have reported that tumor reduction of a stomach leiomyosarcoma was achieved with prolonged treatment using Tegafur [24]. Since the present patient did not want to undergo hospitalized treatment, TS-1 therapy was started on an outpatient basis. During treatment, she reported a loss of appetite that immediately resolved after cessation of treatment. The lung metastasis has been successfully controlled for 17 months with no additional lesions. Thus, prolonged treatment with TS-1 allowed the QOL to be maintained in this patient and possibly extended the survival period.

## 4. Conclusion

A case with a lung metastasis from a perineal leiomyosarcoma was reported. A leiomyosarcoma can reoccur over 5 years from the initial diagnosis; therefore, patients should be monitored for a long period of time. Radical treatment in elder patients with recurrent leiomyosarcoma may result in diminished QOL. The present case may indicate the possibility of TS-1 for inoperable leiomyosarcomas, which permits treatment on an outpatient basis.

## Figures and Tables

**Table 1:** Laboratory data at visitation. Laboratory data showed no abnormalities.

CBC		Biochemistry		Tumor marker	
WBC	4100/*μ*L	AST	18 IU/L	SCC	0.9 ng/mL
RBC	378 × 10^4^/mL	ALT	14 IU/L	SLX	11.6 U/mL
Hb	12.1 g/dL	*γ*-GTP	15 IU/L		
Plt	23.1 × 10^4^/*μ*L	LDH	211 IU/L		
		ALP	186 IU/L		
		TP	7.7 g/dL		
		Alb	4.4 g/dL		
		T.Bil	0.76 mg/dL		
		TG	86 mg/dL		
		HDL	52.6 mg/dL		
		LDL	89 mg/dL		
		BUN	15.2 mg/dL		
		Cre	0.64 mg/dL		
		UA	4.4 mg/dL		
		Na	140 mEq/L		
		K	4.7 mEq/L		
		Cl	104 mEq/L		
		CHE	332 IU/L		
		FBS	107 mg/dL		
		HbA1c	5.8%		

**Table 2:** Lung metastases from a leiomyosarcoma treated by chemotherapy alone over the last 10 years in Japan (2001–2011).

	Sex	Age	Primary lesion	Chemotherapy	Prognosis (after diagnosis of the lung metastasis)	Reference
1	Male	77	Mesocolon transversum	Doxorubicin + ifosfamide	Unknown	[8]
2^†^	Female	39	Uterus	CyVADIC-etoposide	One year and one month	[9]
3^†^	Female	41	Urinary bladder	Dacarbazine + adriamycin + vincristine + cyclophosphamide	One year and three months	[10]
4^†^	Female	55	Uterus	Cisplatin + docetaxel→irinotecan	Two years and three months	[11]
5	Female	48	Uterus	IA→CPT-11→docetaxel + gemcitabine	Eight months	[12]
6	Female	48	Uterus	CAP→IAP	Two years and five months	[13]
7	Female	68	Retroperitoneum	CyVADIC→MAID	Ten months	[14]
8^†^	Female	71	Uterus	Docetaxel + gemcitabine	Two months	[15]
9	Female	49	Uterus	HEEp-DTIC	One year and three months	[16]
10	Female	67	Left adrenal gland	Cisplatin + epirubicin + ifosfamide	One year and seven months	[17]
11^†^	Female	54	Uterus	CyVADIC-etoposide→IAP	Eleven months	[18]
12^†^	Male	44	Spermatic cord	CyVADIC	Five months	[19]

CyVADIC: cyclophosphamide + vincristine + adriamycin + dacarbazine. IA: ifosfamide + adriamycin. CAP: cyclophosphamide + adriamycin + cisplatin. IAP: ifosfamide + adriamycin + cisplatin. MAID: ifosfamide + adriamycin. HEEp-DITC: hydroxycarbamide + epirubicin + etoposide + dacarbazine.

^†^fatal case.
